# A three-timepoint network analysis of Covid-19’s impact on schizotypal traits, paranoia and mental health through loneliness

**DOI:** 10.14324/111.444/ucloe.000044

**Published:** 2022-11-01

**Authors:** Keri Ka-Yee Wong, Yi Wang, Gianluca Esposito, Adrian Raine

**Affiliations:** 1 Department of Psychology and Human Development, University College London, London, UK; 2 Neuropsychology and Applied Cognitive Neuroscience Laboratory, CAS Key Laboratory of Mental Health, Institute of Psychology, Chinese Academy of Sciences, Beijing, China; 3 Department of Psychology, University of Chinese Academy of Sciences, Beijing, China; 4 Department of Psychology and Cognitive Science, University of Trento, Rovereto, Italy; 5 Psychology Program, School of Social Sciences, Nanyang Technological University, Singapore, Singapore; 6 Departments of Criminology, Psychiatry, and Psychology, University of Pennsylvania, Philadelphia, PA, USA

**Keywords:** network analysis, schizotypy, paranoia, anxiety, depression, stress, loneliness, sleep, Covid-19, longitudinal, mental health

## Abstract

The 2019 coronavirus (Covid-19) pandemic has impacted people’s mental wellbeing. Studies to date have examined the prevalence of mental health symptoms (anxiety and depression), yet fewer longitudinal studies have compared across background factors and other psychological variables to identify vulnerable subgroups in the general population. This study tests to what extent higher levels of schizotypal traits and paranoia are associated with mental health variables 6- and 12-months since April 2020. Over 2300 adult volunteers (18–89 years, female = 74.9%) with access to the study link online were recruited from the UK, the USA, Greece and Italy. Self-reported levels of schizotypy, paranoia, anxiety, depression, aggression, loneliness and stress from three timepoints (17 April to 13 July 2020, *N_1_* = 1599; 17 October to 31 January 2021, *N_2_* = 774; and 17 April to 31 July 2021, *N_3_* = 586) were mapped using network analysis and compared across time and background variables (sex, age, income, country). Schizotypal traits and paranoia were positively associated with poorer mental health through loneliness, with no effect of age, sex, income levels, countries and timepoints. Loneliness was the most influential variable across all networks, despite overall reductions in levels of loneliness, schizotypy, paranoia and aggression during the easing of lockdown (time 3). Individuals with higher levels of schizotypal traits/paranoia reported poorer mental health outcomes than individuals in the low-trait groups. Schizotypal traits and paranoia are associated with poor mental health outcomes through self-perceived feelings of loneliness, suggesting that increasing social/community cohesion may improve individuals’ mental wellbeing in the long run.

## Introduction

The coronavirus disease 2019 (Covid-19) pandemic has caused sustained global disruptions to our livelihoods, yet the international scientific community has come together to collect time-sensitive data to shape rapid government responses, policies and vaccine development programmes. Between January 2020 and April 2022, a total of 435,422 publications on coronavirus have been published,^1^ with medical and health sciences being a key area of research interest. Large birth cohort study findings reporting pre- and post-pandemic comparisons – investigating how forced lockdown restrictions have impacted individual’s environments in which they play, work and learn – have been particularly valuable in assessing change. However, many more findings from new cross-sectional cross-country/-population-specific studies have also been pivotal in our understanding of the mental health prevalence under the pandemic conditions. This latter set of studies has often limited the definition of mental health to ‘internalising’ problems such as anxiety and depression, excluding ‘externalising’ problems such as aggression; focused on specific populations (e.g., medical frontline workers, teachers, parents with young children, children with special education needs) and lack control groups. While prevalence rates provide a good ‘snapshot’ of people’s experiences during the pandemic, studies assessing the stability and change of these symptoms in the same individuals throughout the pandemic have been limited due to Covid-19 restrictions, although there are a few exceptions in timeseries studies.^2^ Understanding how environmental factors, such as the imposed national lockdown restrictions (e.g., physical distancing and social isolation), impact mental health [1] is important in identifying groups of individuals who may be more vulnerable and in need of support.

Arguably, a less researched yet important area is the impact of Covid-19 on schizotypal personality traits and paranoia. It is conceivable that Covid-19, an airborne ‘invisible killer’, that has infected over 502 million people – many of whom are asymptomatic – and caused 6.19 million deaths and counting globally,^3^ has instilled doubt and distrust in all aspects of society. We know from existing research that the unfounded fixed belief that others cause intentional harm, or paranoia [2], is a key symptom of mental health disorders and schizophrenia-spectrum disorders such as schizotypal personality disorder – both paranoia and schizotypy exist in varying intensities in the general population [3,4]. For example, as of November 2020, 57% of UK respondents aged 16–75 years (*N* = 2244) expressed distrust in the government’s control over the spread of coronavirus, an increase from 28% at the start of the pandemic in April 2020 [5]. Framing of public health messages which focus on the origin of coronavirus has caused xenophobic aggression towards people of Asian descent [6]. Fear of others not social distancing, fear of catching Covid-19, lack of control over the restrictions and financial uncertainty, are all well-documented stressors that may lead to heightened levels of suspicion towards others and reclusive habits [7]. It is conceivable then that a lockdown will have a bigger effect for individuals with higher levels of paranoia compared to their peers, and higher levels of paranoia may be correlated with mental health issues, including anxiety, depression and aggression, as well as loneliness and Covid-19-related stress.

Compliance with government physical distancing and lockdown restrictions, though necessary in reducing the spread of Covid-19, may perpetuate other health issues. For example, recent studies have shown that lockdown duration (by weeks) can likely increase feelings of loneliness over the course of forced stay-at-home mandates and fuel anxiety and depression [1]. Similarly, higher levels of loneliness are found to be comorbid with other mental health issues in patients with psychosis [8]. Increased fear of one’s and others’ safety, stress about Covid-19 and the lack of social contacts with others may fuel maladaptive thoughts, that if sustained may become paranoia known to be associated with poor psychological wellbeing [9]. In a large representative sample of UK adults in April 2020, mistrust and belief in conspiracy theories were associated with lower compliance in government restrictions, antibody testing and vaccine adoption [10]. Another large study of US adults found that high levels of paranoia are also associated with more endorsements of conspiracy theories generally (e.g., QAnon theories), conspiracies around mask-wearing and potential vaccines [11]. Thus, more than ever, research understanding paranoia and its correlates are of utmost importance in informing public health and policy during the Covid-19 pandemic. From past studies, we also know that paranoia and schizotypal traits are associated with higher levels of anxiety, worries [12], depression [13], insomnia [14,15], loneliness [16] and to a lesser degree, aggression [17,18].

To the best of the authors’ knowledge, four studies have investigated schizotypal personality traits of which paranoia is a key symptom, in relation to mental health during the pandemic. Study findings have been mixed. In one study comparing the UK and Germany adults conducted between 27 April and 31 May 2020, respondents reported experiencing schizotypal traits for the first time (UK = 4.4%, Germany = 3.5%), increases in schizotypal traits (UK = 4.8%, Germany = 4.1%), and a larger group reported unchanged symptom levels (UK = 14.7%, Germany = 14.2%) [19]. No country or gender differences were found despite differences in lockdown restrictions at the time of data collection. By October 2020, the same researchers recruited an additional sample and found that an increase in schizotypal traits was associated with higher levels of loneliness, use of drugs and financial burden, and this was particularly true of UK and not Germany respondents [20]. These changes were thought to be due to sudden changes in environment as a result of national lockdown restrictions and physical distancing measures. In another cross-sectional survey of Tunisian university students conducted between 1 June and 15 July 2020, students self-identified as being in the high schizotypal traits group (top-10% on the 74-item Schizotypal Personality Questionnaire) reported significantly more maladaptive coping strategies and fear of Covid-19 compared to those in the low schizotypal traits group (bottom-10%) [21]. Contrastingly, in an online survey of French adults conducted between 13 April to 11 May 2020 (*N* = 728), paranoia and hallucination were found to be relatively low and associated with cognitive-affective experiences (loneliness, jumping-to-conclusions, anxiety, experiential avoidance), but not associated with Covid-19-related variables (e.g., length of isolation, hospitalisation, Covid-19 symptoms) [22]. While these studies shed light on the mental health correlates with schizotypal traits and paranoia during the pandemic, studies thus far are limited in the scope of mental health variables, follow-up duration and cross-sectional designs which preclude the understanding of specific target variable(s) for intervention and changes in relative associations over time.

One way to fill these gaps is to conduct a network analysis (NA) on all variables and across three timepoints. Mental health variables, such as anxiety, depression and aggression, are often correlated with each other and with schizotypal traits, stress and insomnia, yet traditional bivariate correlations only focus on the association between two variables each time and preclude comparison across interactions and the identification of influential variables in the network. NA addresses this by estimating a network structure, where *‘nodes’* represent the variables and *‘edges’* represent the partial correlations between each pair of variables [23–25]. The ‘*centrality index*’ of nodes reflects the influence of a node in the network and the ‘*strength*’ of the centrality indices is the summed weight of all edges connected to a node in the network, which are important in identifying which variables and relationships are most influential. Mapping the nodes and estimating the edges between pairs of nodes within a network provides a holistic view of all inter-variable relationships and helps identify influential variables for intervention whilst controlling for the effects of all the other variables and associations in the network. Using a network comparison technique, we are able to test invariance of the network structure and strength between variables across networks (age, sex, income, country, timepoints and high vs. low schizotypal trait groups). Furthermore, this study crucially includes a 12-month follow-up at time 3 which allows us to perform the cross-lagged panel network analysis and examine the longitudinal relationships, such as how variables in the previous timepoint predicts a future timepoint of nodes across two timepoints.

This prospective study tests to what extent higher levels of schizotypal traits relate with various mental health variables at 6- and 12-months from April 2020. Three 30-minute online surveys were conducted at three timepoints: 17 April to 13 July 2020 (*N_1_* = 1599), 17 October to 31 January 2021 (*N_2_* = 774) and 17 April to 31 July 2021 (*N_3_* = 586) which coincide with the UK national lockdowns 1, 2 and 3, and the easing of restrictions, respectively. Given the country differences in lockdown restrictions at the time of data collection, we will test to see whether country differences are observed in our outcome variables. As it remains, it is unclear how mental health variables beyond internalising problems, such as externalising problems (aggression), sleep quality and Covid-19-related stressors, relate with schizotypal traits and paranoia over time during the pandemic. Understanding how levels of schizotypal traits and paranoia have varied with both internalising and externalising problems for different groups of individuals (by sex, age, income, country) during the pandemic can help inform government’s rapid response and Covid-19 recover plans, in particular current public health interventions. Using a network analysis, this study tests three hypotheses:

1. Mean levels of schizotypal traits, paranoia and mental health variables will be different across three timepoints, but schizotypal traits and paranoia will be positively associated with poorer mental health symptoms across the three timepoints.
2. The overall network structures will be different for participants across different genders (F = M), age (stronger in <35 vs. 35+ years), countries (stronger in the UK vs. Others), income levels (stronger in low vs. medium vs. high groups) and timepoints (strongest in time 1 > 2 > 3).
3. The network structure will be different for high vs. low paranoid and schizotypal individuals, with associations being stronger for those in the high symptom groups.

## Methods

### Participants

Over 2300 volunteers took part in the survey and were recruited via online advertising of the study, university lists, charity lists, LinkedIn, Twitter, Instagram and word-of-mouth. All adults aged 18 years and above with access to the study website www.GlobalCovidStudy.com could take part. The 30-minute survey hosted online on Qualtrics was available in English and seven other languages (Greek, Italian, Spanish, Chinese Traditional, Chinese Simplified, French, German). Forward translations were first conducted by Google Translate and cross-checked and corrected by at least one native speaker. This study was pre-registered (https://osf.io/4nj3g/ on 17 April 2021) and ethical approval was obtained from the University College London Institute of Education Ethics and Review Committee in April 2020 (REC 1331; [26]). Study preregistration can be found: https://osf.io/fe8q7/. Informed consent was sought from participants at the start of the 30-minute online Qualtrics survey and at subsequent follow-ups, with opt-out options available throughout. Participant demographic and missing data on all study variables across the three timepoints of data collection are presented in Table 1. The analytic sample for this study consisted of data from participants at three timepoints: time 1 (*N_1_* = 1599; 17 April to 14 July 2020), time 2 (*N_2_* = 774; 17 October 2020 to 31 January 2021), and time 3 (*N_3_* = 586; 17 April to 31 July 2021).

**Table 1.** Demographic statistics of all study variables

	Time 1 17 April to 14 July 2020 (*N_1_* = 1599)		Time 2 17 October 2020 to 31 January 2021 (*N_2_* = 774)		Time 3 17 April to 31 July 2021 (*N_3_* = 586)	
	n	%	n	%	n	%
**Age**						
<35 years	952	59.5	446	57.6	339	57.8
35+ years	642	40.2	323	41.7	244	41.6
Missing	5	0.3	5	0.6	3	0.5
**Gender**						
Male	404	25.3	174	22.5	134	22.9
Female	1172	73.3	589	76.1	444	75.8
Else	23	1.4	11	1.4	8	1.4
**Countries**						
UK	649	40.6	360	46.5	281	48
Others	576	36	234	30.2	162	27.6
Missing	374	23.4	180	23.3	143	24.4
**Income**						
Low (<0k)	639	40	281	36.3	179	30.5
Medium (30–60k)	348	21.8	165	21.3	155	26.5
High (>60k)	519	32.5	292	37.7	232	39.6
Missing	93	5.8	36	4.7	20	3.4

### Measures

#### Schizotypal personality traits and paranoia

Schizotypal traits were assessed by the Schizotypal Personality Questionnaire – Brief (SPQ-B; [27]), a 22-item yes/no questionnaire that when summed creates a total score ranging from 0 to 22 with a higher score reflecting more schizotypal traits. Three additional subscales were also created by summing the respective items to form the factors: Cognitive–Perceptual (F1), Interpersonal (F2) and Disorganised (F3) features of schizotypy. The internal reliability for the subscales and total score was good (α = 0.87).

Paranoia was assessed using the Social Mistrust Scale (SMS; [18]), a 12-item 3-point scale (no [0], sometimes [1], yes [2]). Summing all items created a total mistrust score ranging from 0 to 24, whereby a higher score reflected higher levels of paranoia and suspiciousness. Past studies have denoted a score of 7 and above to be ‘mistrustful’. The internal reliability for the total score was good (α = 0.79).

#### Externalising problems

Self-reported levels of aggression were assessed by the Reactive-Proactive Questionnaire (RPQ; [28]), a 23-item self-report questionnaire with a never (0), sometimes (1), often (2) scale. Summing all items produces a total aggression score ranging from 0 to 46 with a higher score reflecting more aggressive behaviours with good internal reliability (α = 0.85).

#### Internalising problems

Depression was assessed using the Patient Health Questionnaire-9 (PHQ-9: [29]), a 9-item 4-point scale (not at all [0], several days [1], more than half the days [2], nearly every day [3]), which when summed produce a total score ranging from 0 to 27. A higher score reflected higher levels of depressive symptoms and a score above 15 was the clinical cut-off. The internal reliability for this study was excellent (α = 0.90).

Anxiety was assessed using the General Anxiety Disorder-7 (GAD-7; [30]), a 7-item 4-point scale (not at all [0], several days [1], more than half the days [2], nearly every day [3]) where a higher summed score across the 7-items ranging from 0 to 21 reflects higher levels of anxiety, with a score above 15 being the clinical cut-off. The internal reliability for this study was excellent (α = 0.92).

#### Feelings of loneliness

The Loneliness Questionnaire (LQ; [31]) is a 20-item (10 reverse-coded items) 4-point scale (never [0], rarely [1], sometimes [2], often [3]) that when summed creates a total score ranging from 20 to 80. A higher score denotes higher levels of loneliness. The internal reliability for this study was excellent (α = 0.94).

#### Sleep quality

Self-reported sleep quality was indexed by summing 4-items from the Consensus Sleep Diary [32] (‘During the past month: How would you rate your overall sleep quality?’, ‘How would you rate the quality of your sleep overall?’ and ‘How rested or refreshed do you feel when you wake up?’) and the Karolinska Sleepiness Scale [33], ‘How sleepy have you felt during the last 5 minutes?’. Scores were summed and range from 4 to 23 with moderate internal reliability (α = 0.66).

#### Covid-19-related stressors

Participants selected from a list of 27 potential stressors related to the Covid-19 pandemic that they thought caused them stress in the past 14 days. Participants were shown a follow-up question with the selected stressors and asked to what extent the following stressors have caused them stress on a 5-point scale: no stress (0), a little bit of stress (1), moderate stress (2), quite a lot of stress (3), extremely stressful (4). Scores were summed and ranged from 0 to 92.

#### Demographic variables

Participants were asked to report on their date of birth (<35 or 35+), gender (female = %), and country at the time of completing the survey (UK vs. Other), which were dichotomised and included in our between-group analyses (see Table 1). Participants reported on their annual pre-tax income in $/£10,000 bands (under £30,000 [0], £30,000–£59,999 [1], £60,000+ [2]), which were categorised and included in our between-group analyses.

### Data analysis

The descriptive statistics of all study variables are reported in Tables 1 and 2 and bivariate relationships are reported in Table 3.

**Table 2.** Descriptive statistics of all variables in network

Time 1	*n*	range	min.	max.	*M*	*SD*	skewness	kurtosis
SPQ-B Total	1599	22	0	22	6.15	4.71	0.73	−0.09
SPQ-B F1	1599	8	0	8	1.73	1.82	1.07	0.55
SPQ-B F2	1599	8	0	8	2.99	2.36	0.44	−0.86
SPQ-B F3	1599	6	0	6	1.43	1.69	1.08	0.14
SMS Total	1599	24	0	24	2.38	2.95	1.90	5.04
RPQ Total	1599	34	0	34	6.74	4.56	1.04	2.02
PHQ-9	1599	27	0	27	7.29	5.60	0.94	0.44
GAD-7	1599	21	0	21	5.60	4.96	1.04	0.40
Stress total	1599	72	0	72	15.24	11.26	1.26	2.12
LQ total	1599	57	20	77	42.49	11.22	0.43	−0.44
Sleep total	1599	19	4	23	12.42	3.69	0.08	−0.57

Time 2	*n*	range	min.	max.	*M*	*SD*	skewness	kurtosis

SPQ-B total	774	21	0	21	5.67	4.82	0.79	−0.16
SPQ-B F1	774	8	0	8	1.50	1.78	1.25	1.04
SPQ-B F2	774	8	0	8	2.88	2.47	0.52	−0.87
SPQ-B F3	774	6	0	6	1.29	1.64	1.20	0.43
SMS Total	774	24	0	24	2.10	2.91	2.29	7.92
RPQ Total	774	24	0	24	4.05	3.97	1.34	2.28
PHQ-9	774	27	0	27	7.14	5.80	1.03	0.58
GAD-7	774	21	0	21	5.56	5.00	1.08	0.55
Stress total	774	92	0	92	15.46	11.41	1.22	2.82
LQ total	774	57	20	77	42.77	11.72	0.41	−0.51
Sleep total	774	18	4	22	13.03	3.67	−0.07	−0.59

Time 3	*n*	range	min.	max.	*M*	*SD*	skewness	kurtosis

SPQ-B total	586	22	0	22	5.35	4.64	0.95	0.39
SPQ-B F1	586	8	0	8	1.32	1.68	1.40	1.49
SPQ-B F2	586	8	0	8	2.83	2.45	0.57	−0.76
SPQ-B F3	586	6	0	6	1.20	1.61	1.34	0.85
SMS total	586	24	0	24	1.90	2.88	2.58	9.59
RPQ total	586	30	0	30	3.60	3.92	2.02	6.56
PHQ-9	586	27	0	27	6.86	5.94	1.33	1.38
GAD-7	586	21	0	21	5.47	5.06	1.22	0.94
Stress total	586	59	0	59	12.95	10.57	1.54	2.54
LQ total	586	55	20	75	41.38	11.81	0.52	−0.26
Sleep total	586	19	4	23	12.81	3.57	0.14	−0.26

Abbreviations: SPQ-B: Schizotypal Personality Questionnaire – Brief; SPQ-B F1: Cognitive-Perceptual; SPQ-B F2: Interpersonal; SPQ-B F3: Disorganised; SMS: Social Mistrust Scale; RPQ: Reactive-Proactive Questionnaire; PHQ-9: Patient Health Questionnaire-9; GAD-7: General Anxiety Disorder-7; LQ: Loneliness Questionnaire.

**Table 3.** Bivariate Pearson’s correlation coefficients between study variables in the network at time 1

	1	2	3	4	5	6	7	8	9	10	11
**1. SPQ-B Total**	–										
**2. SPQ-B F1**	0.765	–									
**3. SPQ-B F2**	0.839	0.413	–								
**4. SPQ-B F3**	0.792	0.479	0.494	–							
**5. SMS Total**	0.453	0.403	0.336	0.358	–						
**6. RPQ Total**	0.335	0.360	0.193	0.276	0.311	–					
**7. PHQ-9**	0.426	0.347	0.350	0.324	0.392	0.278	–				
**8. GAD-7**	0.420	0.396	0.319	0.298	0.354	0.336	0.752	–			
**9. Stress Total**	0.270	0.272	0.203	0.177	0.283	0.256	0.565	0.595	–		
**10. LQ Total**	0.610	0.365	0.619	0.442	0.502	0.243	0.539	0.453	0.320	–	
**11. Sleep Total**	0.240	0.187	0.204	0.182	0.238	0.137	0.558	0.454	0.352	0.338	–

Abbreviations: SPQ-B: Schizotypal Personality Questionnaire – Brief; SPQ-B F1: Cognitive-Perceptual; SPQ-B F2: Interpersonal; SPQ-B F3: Disorganised; SMS: Social Mistrust Scale; RPQ: Reactive-Proactive Questionnaire; PHQ-9: Patient Health Questionnaire-9; GAD-7: General Anxiety Disorder-7; LQ: Loneliness Questionnaire.

**Group comparison.** Independent sample *t*-tests were conducted to examine the group differences between age (older vs. younger), gender, country (UK vs. other counties) and socioeconomic status (low, medium, high). Paired sample *t*-tests were conducted to examine the changes in all psychological variables between two timepoints. SPSS 19.0 was for aforementioned statistics with significant threshold set at *p* < 0.05.

**Network estimation.** Psychological networks were estimated in the whole sample collected at the first timepoint to examine the direct links between psychological variables including anxiety (GAD), depression (PHQ), sleep, Covid-19-related levels of stress, loneliness (Lone), aggressions (RPQ), paranoia (SMS) and the three factors of the schizotypy subscales (SPQ-B: Factor 1, 2, 3). In this study, nodes were defined as participants’ scores on each of the variables and edges were calculated using partial correlations between pairs of nodes after controlling for all the other variables in the network. The Graphical Least Absolute Shrinkage and Selection Operator (LASSO) [34] in combination with the Extended Bayesian Information Criteria (EBIC) model selection [35] were used to estimate the Gaussian graphical model and to construct networks. Furthermore, to investigate the importance of each node in the network, the strength of each node was examined by summing up all connections of the node. Out of all the centrality indices, we mainly report on the index of ‘*strength*’ as all connections are positive and the nodes are total or subscale scores of psychological variables. The standardised z-scores of centrality indices were calculated and reported. The ‘*bootnet*’ package (https://CRAN.R-project.org/package=bootnet) implemented in R statistical software (version 4.0.2, https://www.r-project.org/) were used to construct the networks and the ‘*qgraph*’ package (https://CRAN.R-project.org/package=qgraph) was used for centrality calculation and visualisation. The force-directed Fruchterman–Reingold algorithm [36] was used to determine the placement of nodes in the network and how they are estimated in the sample.

**Network Comparison Test (NCT)**. The ‘Network Comparison Test’ package (https://CRAN.R-project.org/package=NetworkComparisonTest) was used to examine the invariance of two networks. The tests of network invariance usually include invariance of network *structure*, *global strength* and *edge* weights of the network. In order to compare the networks between the groups by age, gender, countries and income levels, as well as individuals with high and low schizotypal traits, we estimated networks for each subset of data and then conducted the NCT, respectively, using two-tailed permutation tests at 10,000 times [37]. To cater for multiple comparisons of invariance tests of edge-weights and nodal strength, false discovery rate (FDR) correction was applied to correct for the estimations. The significance threshold was set at *p* or adjusted *p* < 0.05.

**Cross-lagged panel network (CLPN) analysis.** Longitudinal relationships of nodes were estimated using CLPN modelling [38]. As there are three timepoints, we performed CLPN analysis separately for two timepoints at a time, to examine which variables in the earlier timepoint were most predictive of the variables at the later timepoint (e.g., predict variables at time 2 based on time 1). The CLPN was estimated using a series of nodewise linear regression models to compute autoregressive (i.e., the coefficient of a node at time 1 predicts itself at time 2 after controlling for all other nodes at time 1) and cross-lagged effects (i.e., the coefficient of a node at time 1 predicts another node at time 2 after controlling for all the other nodes at time 1). Regression coefficients were regularised using LASSO with 10-fold cross-validation tuning parameter selection to shrink small regression coefficients to exactly zero. Regularised regressions were estimated using ‘*glmnet*’ package in R (https://CRAN.R-project.org/package=glmnet).

**Network stability and accuracy**. The stability and accuracy of each estimated network were examined with reference to a tutorial paper by Epskamp et al. [39] (see Supplementary Figs S1–S8, Figs S1–S4 for wave 1, S5–S6 for wave 2 and S7–S8 for wave 3).

## Results

### Descriptive statistics

Descriptive statistics of study variables (Tables 1 and 2) and bivariate correlations of all study variables are presented below (Table 3). All correlation coefficients were statistically significant and positively correlated with each other at *p* < 0.001 level. Comparison of proportions using https://www.medcalc.org/calc/comparison_of_proportions.php [40] found no significant differences in participants in time 1 and 3 on age, gender and income (*p* > 0.1).

### Comparisons of all study variables across age, gender, countries and income groups at time 1

Independent samples *t*-tests were conducted to test for groups differences between younger and older groups, males and females, countries (UK vs. Other countries) as well as socioeconomic status. In addition, multivariate analysis of variance (MANOVA) was conducted to compare groups with different levels of income. Adjusted *p* (0.05/11 = 0.0045) was considered as a significance threshold to correct multiple comparisons. The results in detail were shown in Table 4.

**Table 4.** Comparisons across age, gender, countries and income groups at time 1

Time 1	Age		Gender		Countries		Levels of income		
	Younger vs. older		Male vs. female		UK vs. Others		(Low vs. medium vs. high)		
	*t*	*p*	*t*	*p*	*t*	*p*	*p*	*p*	Post hoc
SPQ-B total	**4.47**	<0.001	2.00	0.045	**2.94**	0.003	**30.52**	<0.001	L > M > H
SPQ-B F1	**3.16**	0.002	−0.62	0.537	0.78	0.437	**21.14**	<0.001	L > M > H
SPQ-B F2	**3.09**	0.002	1.06	0.289	**3.50**	<0.001	**18.87**	<0.001	L = M > H
SPQ-B F3	**4.84**	<0.001	**4.53**	<0.001	2.41	0.016	**21.27**	<0.001	L > M > H
SMS total	−1.28	0.201	1.51	0.131	0.40	0.691	**29.15**	<0.001	L > M > H
RPQ total	**3.22**	0.001	−0.69	0.493	−2.84	0.005	**21.96**	<0.001	L > M = H
PHQ-9	**6.31**	<0.001	−**4.65**	<0.001	**6.13**	<0.001	**18.00**	<0.001	L = M > H
GAD-7	**5.79**	<0.001	−**6.98**	<0.001	**4.18**	<0.001	**9.09**	<0.001	L = M > H
Stress total	**5.71**	<0.001	−**5.00**	<0.001	**3.00**	0.003	**16.20**	<0.001	L > M > H
LQ total	0.87	0.383	1.08	0.279	**3.80**	<0.001	**16.23**	<0.001	L = M > H
Sleep total	**2.91**	0.004	−2.41	0.016	**4.84**	<0.001	0.50	0.606	–

Abbreviations: SPQ-B: Schizotypal Personality Questionnaire – Brief; SPQ-B F1: Cognitive-Perceptual; SPQ-B F2: Interpersonal; SPQ-B F3: Disorganised; SMS: Social Mistrust Scale; RPQ: Reactive-Proactive Questionnaire; PHQ-9: Patient Health Questionnaire-9; GAD-7: General Anxiety Disorder-7; LQ: Loneliness Questionnaire. *p* < 0.0045 (0.05/11) was set as threshold to adjust for multiple comparisons.

Numbers in bold indicate significant group differences after multiple comparisons.

In summary, the younger group (<35 years) reported higher levels of schizotypal traits, aggression, depression, stress and anxiety, as well as more sleep problems compared to older participants (35+ years); females reported more severe depression, stress and anxiety than male participants. Compared to the other countries, participants from the UK had higher levels of schizotypal traits, depression, anxiety, loneliness and sleep problems, and lower aggressive behaviours. High income was a protective factor for schizotypal traits, negative affect and loneliness compared to the individuals in the medium- or low-income bands.

### Comparisons of all study variables across time

To examine the changes across time, we conducted paired samples *t*-tests on all study variables between time 1 and 2, as well as between time 2 and 3, respectively. The results suggested that participants reported lower levels of aggressive behaviours and more sleep problems at time 2 compared to time 1. In the last timepoint, participants had lower levels of schizotypal traits and stress caused by Covid-19. These changes were significant even after multiple comparison corrections with adjusted *p* < 0.0045 were applied.

### Network analysis: network estimation and inference in the whole sample of time 1

**Figure 1:**
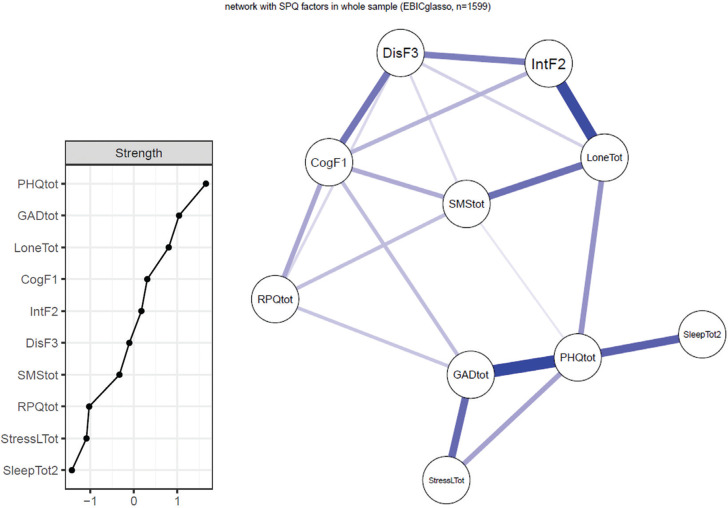
Estimated network structure of time 1 using SPQ factor scores (right) and nodal strength (left). All of the blue lines in the network represent positive partial correlations. A thicker line represents a stronger correlation. Abbreviations: SPQ-B: Schizotypal Personality Questionnaire – Brief, SMStot: Social Mistrust Scale, RPQtot: Reactive-Proactive Questionnaire, PHQtot: Patient Health Questionnaire-9, GADtot: General Anxiety Disorder-7, LoneTot: Loneliness Questionnaire, StressLTot: Covid-19-related stressors, SleepTot2: self-reported sleep quality, CogF1: Cognitive-Perceptual factor of SPQ-B, IntF2: Interpersonal factor of SPQ-B, DisF3: Disorganized factor of SPQ-B.

In the whole sample of time 1, we estimated a network using all study variables including three factors of the SPQ-B, shown in Fig. 1. The line between a pair of variables indicates the partial correlations after controlling for all other variables in the network, with thicker lines representing stronger bivariate connections. Strong connections were observed between schizotypal traits, paranoia and mental health variables. For example, SPQ-B Factor 1 was linked with anxiety, aggression and paranoia, while SPQ-B Factor 2 was correlated with depression through loneliness.

Figure 1 details the strength of all study variables from time 1. Depression, anxiety and loneliness are seen to be the most influential nodes in the network as they have relatively high nodal strength. According to the network, anxiety, depression and stress from Covid-19 were closely correlated to each other, while sleep problems were only connected with depression. More interestingly, we found that loneliness was connected to multiple nodes in the network, including schizotypal traits (SPQ-B Factor 2 and Factor 3), paranoia and depression. This finding suggests that loneliness may serve as a bridge connecting schizotypal traits/paranoia with poor mental health.

### Network comparisons test (NCT) across groups

At time 1, network comparisons were conducted across groups by age (<35 years, 35+ years), gender (male vs. female), countries (UK vs. Others) and levels of income (low, medium, high). NCT analyses did not show significant differences in network structures or global strength between **age groups** (younger vs. older groups, network structure invariance test: *M* = 0.12, *p* = 0.243; global strength invariance: 3.86 for younger group and 4.04 for older group, *S* = 0.18, *p* = 0.106, global strength for network of younger group is 3.86 and 4.04 for the network of older group). Given differences in sample sizes, we repeated the NCT 100 times using random subsamples of younger participants and found that only 1% and 16% of the invariance tests for network and global strength were found to be significant – confirming our null finding. No **gender differences** were found between males and females (network structure: *M* = 0.12, *p* = 0.448; global strength: *S* = 0.16, *p* = 0.196, global strength for the network of males is 3.86 and 4.02 for females). Repeated subsampling and NCT showed that only 13% and 3% in invariance tests of the network structure and global strength were significant, respectively – again confirming null finding. In terms of **networks of UK and other countries**’ responses, again, no significant differences were found no matter on network structure (*M* = 0.15, *p* = 0.170) or global strength (*S* = 0.07, *p* = 0.610, global strength for the network of UK participants is 3.98 and 3.91 for others). Comparing networks across groups with **low, medium and high levels of income** also resulted in no significant differences (low vs. medium income group: network structure: *M* = 0.14, *p* = 0.300; global strength: *S* = 0.07, *p* = 0.647; low vs. high income group: network structure: *M* = 0.13, *p* = 0.335; global strength: *S* = 0.06, *p* = 0.570; medium vs. high income group: network structure: *M* = 0.23, *p* < 0.05; global strength: *S* = 0.003, *p* = 0.984). These findings indicated that networks were comparable (i.e., invariant) across different groups: age, gender, countries and levels of income.

**Figure 2:**
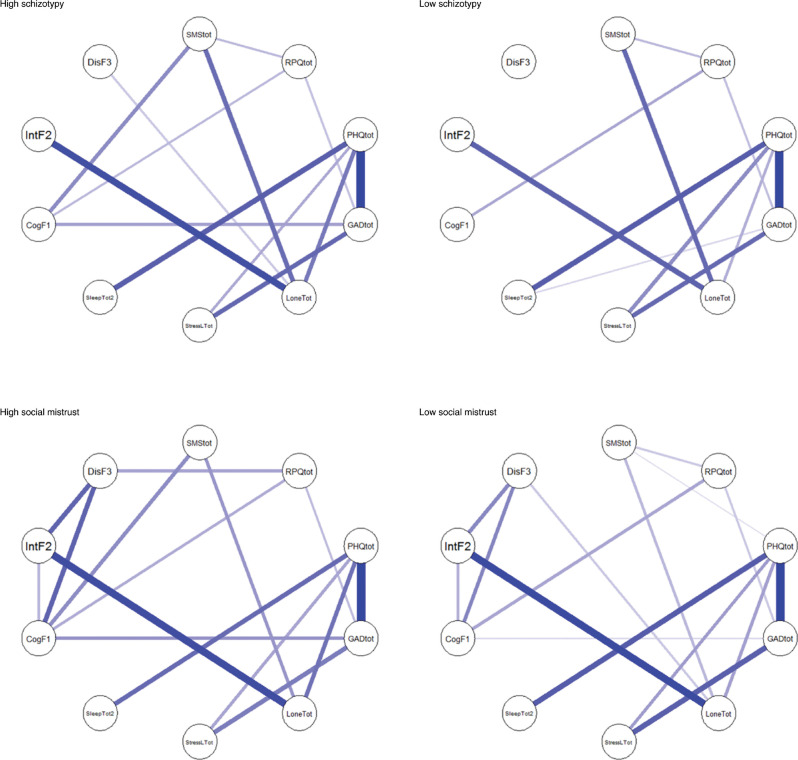
Networks of all study variables by high-/low-schizotypy groups (top) and high-/low-paranoia groups (bottom).

Furthermore, we also performed network comparisons between **high vs. low schizotypy/paranoia** groups. The network structures between groups with high and low SPQ-B scores were different (*M* = 0.21, *p* < 0.001). Compared with the low schizotypy group, individuals in the high schizotypy group showed significantly stronger correlations between paranoia and SPQ-B Factor 1 (adjusted *p* = 0.005), anxiety and SPQ-B Factor 1 (adjusted *p* = 0.027), and loneliness and SPQ-B Factor 2 (adjusted *p* < 0.001). The global strength of the high schizotypy group was also stronger than the low schizotypy group (*S* = 1.10, *p* < 0.001, 2.66 for low SPQ group and 3.76 for high SPQ group). In terms of the paranoia, individuals in the high paranoia group also showed a different network structure compared with those in the low paranoia group (*M* = 0.183, *p* = 0.004). Stronger network connections were found between paranoia and SPQ-B Factor 1 (adjusted *p* < 0.05) and loneliness (adjusted *p* < 0.001) in the high SMS group compared with the low SMS group. The global strength for the high SMS group was significantly higher than that of the low SMS group, 3.82 vs. 3.30, respectively (*S* = 0.53, *p* < 0.05, see networks in Fig. 2).

### Network comparisons across timepoints and longitudinal relationships

**Figure 3:**
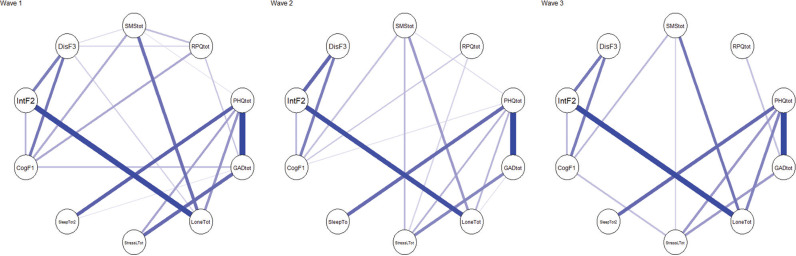
Invariance test of network structures across three timepoints.

We performed the network comparisons to test the invariance of network structure and global strength across three timepoints with each other (Fig. 3). Compared to the time 1 network, the time 2 network had comparable network structure (*M* = 0.11, *p* = 0.153) and global strength (*S* = 0.02, *p* = 0.879, 3.99 for time 1 and 4.02 for time 2), suggesting that no significant differences in the networks were found across two timepoints. Similarly, the networks of time 2 and time 3 are similar with no significant differences (*M* = 0.08, *p* = 0.983; *S* = 0.07, *p* = 0.519, global strength is 4.02 for time 2 and 3.95 for time 3). These findings indicate that network structure and partial correlations among variables were similar across the three timepoints.

**Figure 4:**
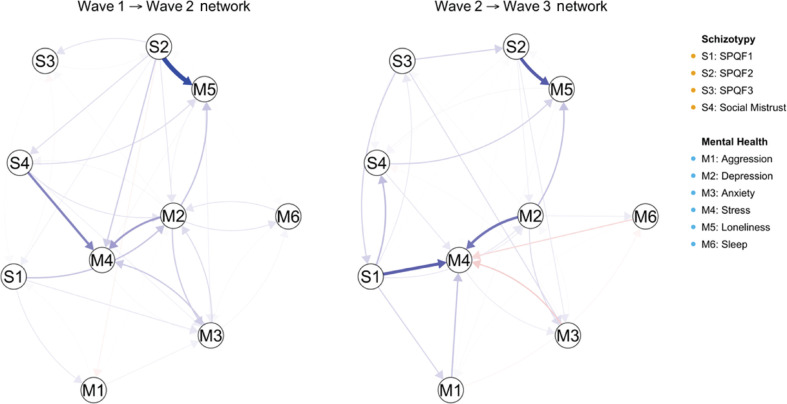
The results of cross-lagged panel network (CLPN) analysis. Arrows represent unique longitudinal relationships which were calculated by the regression analysis across data from different timepoints. Blue edges indicate positive relationships, and red edges indicate negative relationships. Thicker edges represent stronger relations. Autoregressive edges were excluded.

The results of CLPN are shown in Fig. 4. The SPQ-F2 and social mistrust at time 1 could predict the scores on stress and loneliness, respectively, at time 2, while SPQ-F2, SPQ-F1 and depression at time 2 predicted the loneliness and stress at time 3. All these cross-lagged effects survived after applying a threshold of 0.35.

## Discussion

### Main findings

In this three-timepoint network analytic study of the associations between paranoia and schizotypal traits in relation to anxiety, depression, loneliness, aggression, Covid-19-related stress and poor sleep, we found that both paranoia and schizotypal traits were positively associated with depression and associated relationships with anxiety, stress and poor sleep primarily through self-perceived loneliness. Specifically, interpersonal and disorganised features were particularly associated with loneliness and depression – a key relationship observed in individuals in the high-schizotypy and high-paranoia group but not the low-trait groups – while cognitive–perceptual features of schizotypy were specifically associated with anxiety. Both paranoia and schizotypal traits were uniquely associated with aggression. Interestingly, there were no network structure differences across sex, age groups, countries and income level, indicating that no single vulnerable group could be identified but rather the effects were similar on the whole. On the contrary, we found significant differences in network structure between high and low schizotypal traits/social mistrust groups, with high schizotypal trait/mistrust groups showing stronger network connections compared to their counterparts. Between time 1 and 2, there was a reduction in schizotypal traits and aggression, but an increase in poor sleep for the same participants. Between time 2 and 3, there was an overall reduction in levels of Covid-19-related stress, schizotypal traits, aggression, paranoia and loneliness. This is in line with the changes in country lockdown restrictions at the time – in the UK, the US, Italy and Greece where the majority of respondents contributed from – lockdown restrictions were easing, shops were reopening, and physical distancing was still in place but group gathering limitations were being lifted. On balance, these findings tentatively suggest that reductions in self-perceived loneliness – an influential variable across all participant groups – may have taken place due to the improved environmental situation during the easing of lockdown, and this in turn may have reduced concurrent negative associations between paranoia/schizotypy and mental health symptoms to a large degree.

Although the empirical evidence for why schizotypal traits are associated with loneliness remains sparse, it is conceivable that individuals with schizotypy feel anxious in social situations (F2), often have few close friends and anhedonia, and these in turn are features that may also prevent other people from interacting with the individual and precipitate feelings of loneliness. Indeed, a large-scale meta-analytic study has documented a moderate effect between loneliness and schizotypal traits [*N* = 15,647; *k* =13, *r* = 0.32, 95% confidence interval [CI] (0.20 – 0.44)] [41], with effects replicated for both positive and negative symptoms of schizotypy [42]. Conversely, these study findings are also consistent with studies of first-episode schizophrenia patients who report having more days during the week in which they feel lonely, perhaps associated with the poorer social network and support, and associated symptoms of depression and anxiety [43]. Another explanation for this relationship could be that the fear of others causing harm or paranoia, coupled with an individual’s odd behaviours and social anxiety, can lead an individual to keep to themselves more or avoid social situations altogether, which in turn can lead to reduced interactions with others and can spiral into a vicious cycle where alternative positive interactions are not possible, and self-perceived detachment from others and loneliness ensues. Whether the causal direction of changes in levels of schizotypy and paranoia are purely due to the easing of Covid-19 restrictions taking place during time 3 (April to July 2021), natural acclimatisation to the pandemic and/or existing poor social support/earlier childhood experiences may be disputed, as we do not have pre-pandemic baseline measures of paranoia. Drawing on developmental research comparing suspicious and non-suspicious children, highly suspicious 9–16-year-olds were more likely to report feelings of loneliness, more negative peer relationships, such as being victims of bullying, and a hostile attributional style of thinking about others [44], suggesting that negative changes in an environment may also be a cause of the loneliness and schizotypy/paranoia relationship.

Over a 12-month period (time 1 and time 3), schizotypal traits and paranoid ideations have reduced over time, yet we only see reductions in levels of loneliness between time 2 and 3 (*p* < 0.002) synced with easing of lockdown, and not between time 1 and time 2 (*p* = 0.273) (see Table 5). Two explanations may account for this: first, levels of loneliness were generally felt and sustained for the large majority of the sample given that the UK was in full national lockdowns coinciding with time 1 and time 2 data collection, uncertainty around coronavirus was high, and worldwide travel restrictions were in place. For most people, this unprecedented forced separation from the world was a first. By time 3, mean levels of self-perceived loneliness reduced, which coincided with the initial easing of lockdown restrictions (e.g., reopening of shops, going out was possible, yet physical distancing 2-metre rule was still in place until the end of time 3 data collection 19 July 2021). Unfortunately, without a fourth time point, it is not possible to see whether levels of loneliness continue to stabilise or decline to pre-pandemic levels. Perhaps unsurprisingly, initial easing with certain restrictions still in place (e.g., limited numbers for gatherings, working from home, shops not fully open, vaccine rollout at 90%) was helping reduce feelings of loneliness for the majority of respondents. This is consistent with a small experimental study of community samples (*N* = 60), whereby using a false-feedback paradigm to manipulate feelings of loneliness have been shown to lead to decreases in paranoid beliefs [45]. This finding perhaps suggests that government and community efforts to reduce feelings of loneliness may be beneficial for the large majority of the general public.

**Table 5.** Comparisons of all study variables across time using paired samples t-tests

	T1 vs. T2					T2 vs. T3				
	mean diff.	SD	*t*	*df*	*p*	mean diff.	SD	*t*	*df*	*p*
SPQ-B total	0.36	3.00	**3.09**	672	0.002	0.23	2.40	2.00	435	0.046
SPQ-B F1	0.05	1.26	1.10	672	0.272	0.18	1.14	**3.32**	435	0.001
SPQ-B F2	0.16	1.58	2.59	672	0.010	−0.03	1.46	−0.49	435	0.622
SPQ-B F3	0.15	1.29	**2.94**	672	0.003	0.08	1.05	1.64	435	0.101
SMS total	0.10	2.38	1.08	672	0.279	0.25	2.26	2.27	435	0.024
RPQ total	2.42	3.89	**16.17**	672	<0.001	0.37	3.20	2.38	435	0.018
PHQ-9	0.15	4.33	0.87	672	0.383	0.16	4.30	0.77	435	0.443
GAD-7	−0.02	4.10	−0.12	672	0.903	−0.07	4.22	−0.35	435	0.725
Stress total	0.24	8.85	0.69	672	0.492	2.19	8.39	**5.46**	435	<0.001
LQ total	−0.31	7.27	−1.10	672	0.273	1.07	7.29	**3.08**	435	0.002
Sleep total	−0.56	3.53	−**4.13**	672	<0.001	0.20	3.16	1.29	435	0.199

Abbreviations: SPQ-B: Schizotypal Personality Questionnaire – Brief; SPQ-B F1: Cognitive-Perceptual; SPQ-B F2: Interpersonal; SPQ-B F3: Disorganised; SMS: Social Mistrust Scale; RPQ: Reactive-Proactive Questionnaire; PHQ-9: Patient Health Questionnaire-9; GAD-7: General Anxiety Disorder-7; LQ: Loneliness Questionnaire. *p* < 0.0045 (0.05/11) was set as threshold to adjust for multiple comparisons.

Numbers in bold indicate significant group differences after multiple comparisons.

A second explanation for the evolution of self-perceived levels of loneliness observed in our study is based on individual differences. Participants responded to the survey at different times of the lockdown period, and our assessment at 6 and 12 months may have been too long to capture smaller in-person fluctuations. As we know from our time 1 findings that the levels of self-perceived loneliness follow an inverted U-shape in relation to lockdown duration in weeks: respondents to the survey at the beginning and end of the lockdown period reported significantly higher mean levels of loneliness compared to those in the middle weeks of the lockdown period [1]. This may suggest that there are individual differences variations on self-perceived levels of loneliness (but not for other mental health variables) as lockdown duration progresses, perhaps alternative factors that we have not assessed in this study that may come into play including an individual’s ability to cope and access financial and emotional support during the lockdown period [21]. Thus, future studies using latent class analysis to identify high vs. low levels of loneliness groups in relation to differences in mental health and schizotypal traits may help clarify the role of loneliness in this network analysis.

Controlling for other variables in the network, study network analyses failed to find network structure differences across groups, suggesting that for all groups, loneliness is a key variable through which paranoid ideations and schizotypal traits are associated with heightened levels of mental health issues and symptoms (e.g., depression, anxiety, poor sleep, Covid-19-related stress). This finding is consistent with previous studies showing that reductions in loneliness through weekly positive psychology interventions or social prescribing can improve psychological wellbeing for older adults [46] and patients with psychosis [47], and increase neighbourhoods’ identification and social belonging [48]. Thus, investing in community services that prevent social isolation as part of the pandemic recovery strategy may be key in reducing feelings of loneliness for the general population [49]. When splitting all participants at time 1 into high and low schizotypy/paranoia groups, we observed a stronger connected network for the high schizotypy/paranoia group compared to individuals with low level of schizotypy/paranoia. This is consistent with our expectation, as the network theory [50] assumes that individuals with severe symptoms would have more nodes activated and manifest a stronger connected network. Hence, it is very important to identify some nodes in the network which would be greatly influential to the other variables and relatively easy to manipulate. Based on our findings, loneliness would be a promising node that could be consider for future intervention.

As most published findings focus primarily on internalising problems and not externalising problems – a key gap addressed in this study – the finding that paranoia/schizotypy uniquely relate to aggression highlights the importance of assessing comorbid psychopathology [51]. The schizotypy–aggression relationship observed in this study is consistent with prior pre-pandemic literature [52,53], indicating that above and beyond the mental health variables included in the network, schizotypal traits were associated with more aggressive behaviours, specifically reactive retaliatory aggression and not proactive instrumental aggression. This suggests that individuals with high schizotypal traits are more likely to report retaliatory aggression as a result of social interactions with others (not proactive aggression), and thus are more likely to perhaps avoid social situations, engage in reclusive behaviours and report higher feelings of loneliness than individuals in the low-trait group. Particular attention to helping individuals with high levels of paranoia and schizotypy reintegrate into communities post-pandemic may be warranted.

### Strengths and limitations

This study begins to answer how schizotypal traits and paranoid ideations are associated with various mental health variables for different groups of individuals during the pandemic year. To the best of the authors’ knowledge, this is also the first study to explore both schizotypal traits and paranoia together and internalising and externalising symptoms using a network analytic approach to identify the variable(s) of influence for intervention and across a 12-month period during the pandemic. Our study was able to examine macro and micro associations, test for group contrasts and across timepoints that coincided with national lockdowns and easing periods in the UK and to a large extent, abroad as well. This analytic technique, although not commonly used across timepoints, may be particularly valuable when applied to big data to glean a holistic understanding of the web of comorbid relationships that are often observed in mental health research.

This study is however, not without limitations. First, our participants were recruited online via convenience sampling and may not be generalisable to the population of each country where sample size remained relatively small – although this time-sensitive data may still be helpful where future comparative studies with international groups with the same measures are possible. Second, those who chose to take part were particularly willing and had access to technology to complete the survey online, thus potentially they are of a more affluent and motivated group. However, the median income reported by our sample shows that 50% are under £30,000, which is similar to the UK national average for 2021, £31,460 [54]. Third and finally, our survey relies on self-reporting, which would suggest that the associations between variables are inflated, although arguably self-reporting is the most valid and appropriate method of design given the Covid-19 pandemic restrictions. Nonetheless, these study findings spanning the 12-month pandemic period following the same participants do replicate pre-pandemic findings in the literature, specifically highlighting loneliness as a key variable for intervention for governments and local communities in the Covid-19 recovery plans to improve people’s psychological and relational health.

## Figures and Tables

**Table S1.** Correlation coefficients between each pair of variables in network of time 2

	0	1	2	3	4	5	6	7	8	9	10
**0. SPQ-B total**	1										
**1. SPQ-B Factor1**	0.762**	1									
**2. SPQ-B Factor2**	0.865**	0.437**	1								
**3. SPQ-B Factor3**	0.811**	0.496**	0.563**	1							
**4. SMS**	0.424**	0.380**	0.323**	0.348**	1						
**5. RPQ total**	0.160**	0.218**	00.059	0.144**	0.201**	1					
**6. PHQ total**	0.467**	0.401**	0.382**	0.362**	0.467**	0.172**	1				
**7. GAD total**	0.420**	0.374**	0.338**	0.321**	0.432**	0.215**	0.789**	1			
**8. Stress total**	0.378**	0.343**	0.301**	0.285**	0.446**	0.233**	0.623**	0.632**	1		
**9. Loneliness total**	0.610**	0.358**	0.635**	0.450**	0.487**	0.150**	0.569**	0.514**	0.453**	1	
**10. Sleep total**	0.274**	0.215**	0.235**	0.218**	0.256**	0.082*	0.559**	0.452**	0.387**	0.356**	1

Abbreviations: SPQ-B: Schizotypal Personality Questionnaire – Brief, SMS: Social Mistrust Scale, RPQ: Reactive-Proactive Questionnaire, PHQ: Patient Health Questionnaire-9, GAD: General Anxiety Disorder-7.

**p < 0.01, *p < 0.05.

**Table S2.** Correlation coefficients between each pair of variables in network of time 3

	0	1	2	3	4	5	6	7	8	9	10
**0. SPQ-B total**	1										
**1. SPQ-B Factor1**	0.759**	1									
**2. SPQ-B Factor2**	0.862**	0.444**	1								
**3. SPQ-B Factor3**	0.780**	0.470**	0.499**	1							
**4. SMS**	0.480**	0.421**	0.387**	0.355**	1						
**5. RPQ total**	0.281**	0.272**	0.225**	0.186**	0.310**	1					
**6. PHQ total**	0.478**	0.399**	0.405**	0.347**	0.462**	0.315**	1				
**7. GAD total**	0.447**	0.357**	0.392**	0.320**	0.429**	0.351**	0.772**	1			
**8. Stress total**	0.408**	0.397**	0.323**	0.270**	0.428**	0.319**	0.633**	0.610**	1		
**9. Loneliness total**	0.636**	0.408**	0.653**	0.414**	0.556**	0.289**	0.609**	0.517**	0.480**	1	
**10. Sleep total**	0.202**	0.145**	0.185**	0.149**	0.181**	0.137**	0.516**	0.416**	0.357**	0.296**	1

Abbreviations: SPQ-B: Schizotypal Personality Questionnaire – Brief, SMS: Social Mistrust Scale, RPQ: Reactive-Proactive Questionnaire, PHQ: Patient Health Questionnaire-9, GAD: General Anxiety Disorder-7.

**p < 0.01.

**Figure S1:**
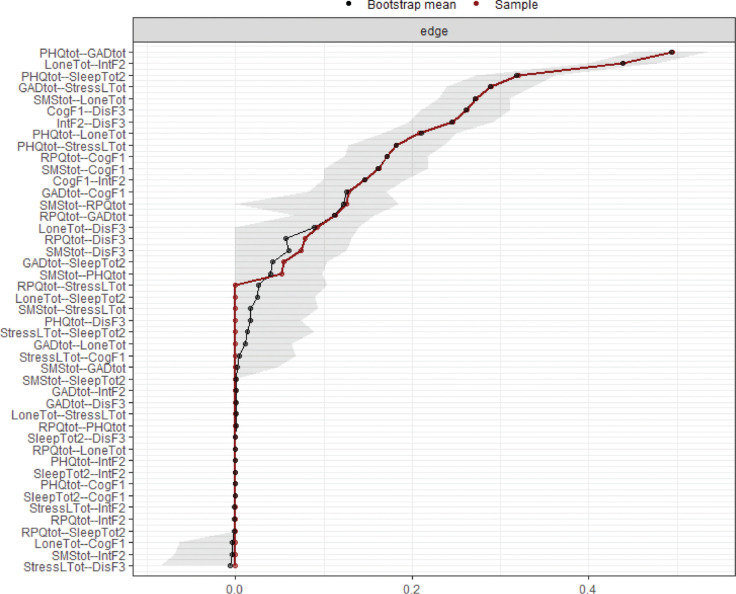
Bootstrapped CIs of estimated edge-weights for the estimated network. The red line indicates the sample values and the grey area indicates the bootstrapped CIs. Each horizontal line represents one edge of the network ordered by edge-weights.

**Figure S2:**
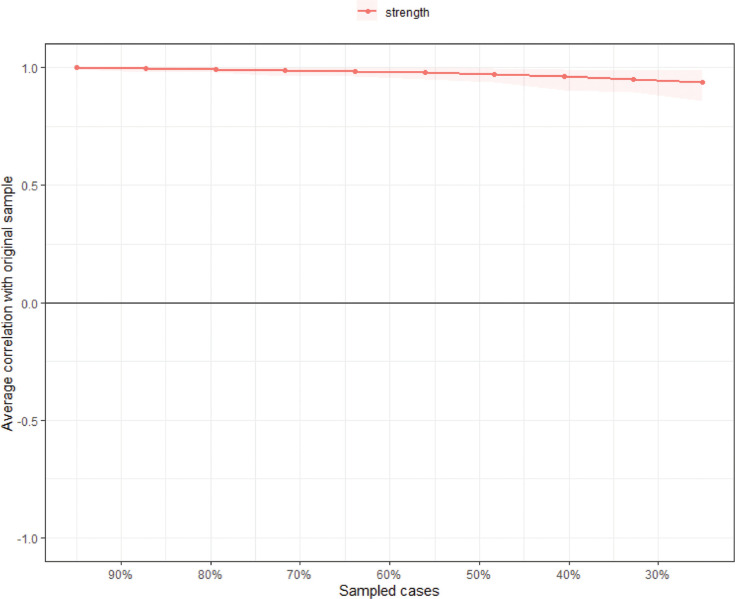
Average correlations between strengths of networks estimated with sampled participants and original sample. Lines indicate the means and areas indicate the range from the 2.5th to the 97.5th percentile.

**Figure S3:**
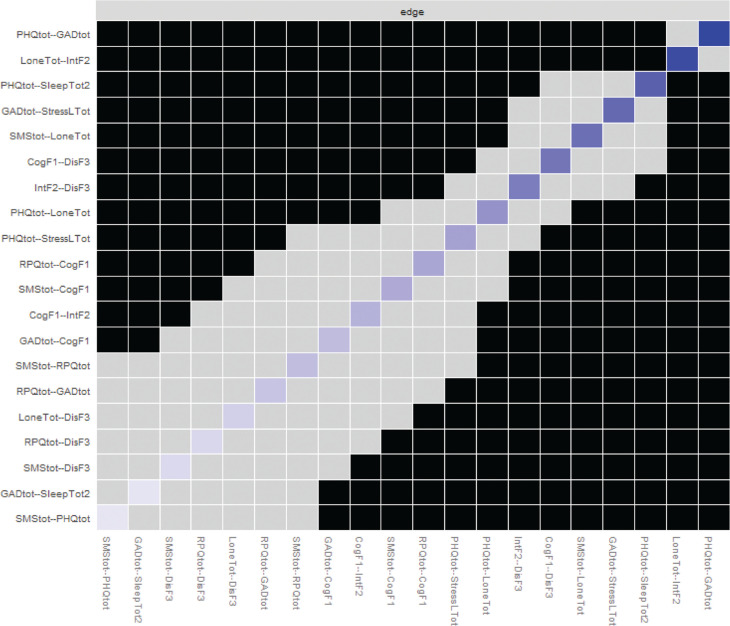
Bootstrapped difference tests on the non-zero edge-weights of the estimated network. Black boxes indicate edges that differ significantly from other corresponding edges in the matrix. Coloured boxes in the edge-weight plot correspond to the colour of edges in the estimated network.

**Figure S4:**
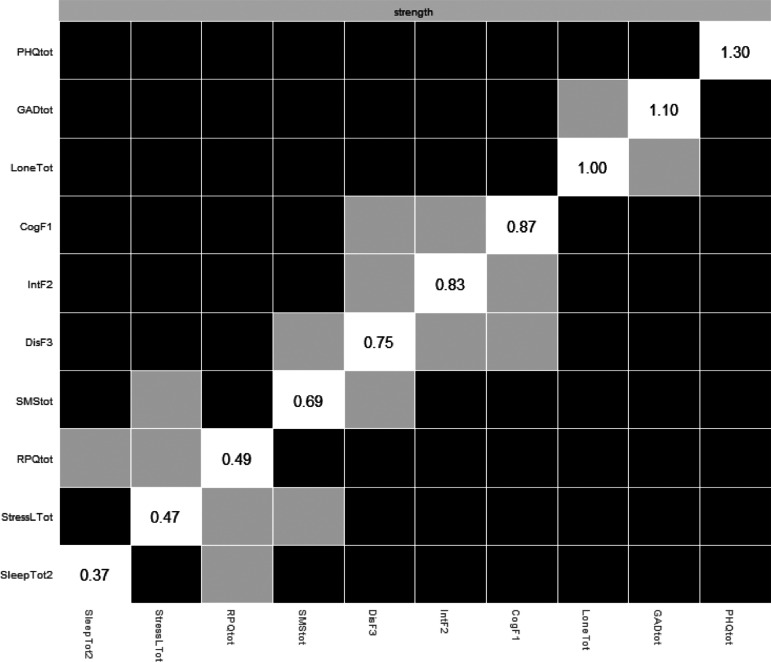
Bootstrapped difference tests on the nodal strength of all the variables in the network. Black boxes indicate nodes that differed significantly from another corresponding node in the matrix. Numbers in white boxes in the centrality plot show the strength of the corresponding node.

**Figure S5:**
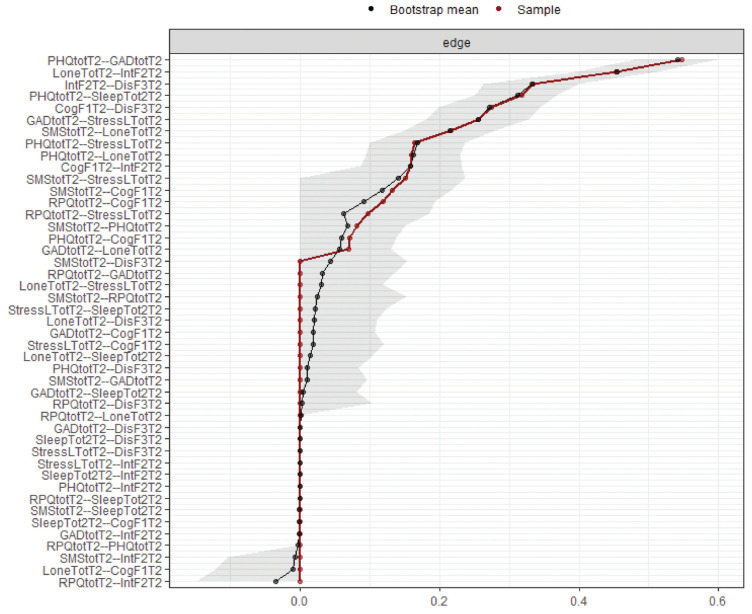
Bootstrapped CIs of estimated edge-weights for the estimated network at time 2. The red line indicates the sample values and the grey area indicates the bootstrapped CIs. Each horizontal line represents one edge of the network ordered by edge-weights.

**Figure S6:**
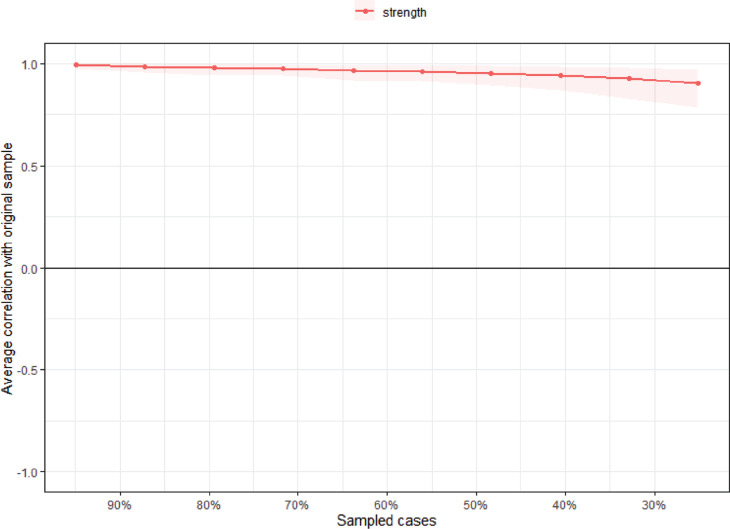
Average correlations between strengths of networks estimated for time 2 with sampled participants and original sample. Lines indicate the means and areas indicate the range from the 2.5th to the 97.5th percentile. The CS-coefficient for strength [CS (cor = 0.7) = 0.749] is higher than 0.5 suggesting the centrality indices were stable.

**Figure S7:**
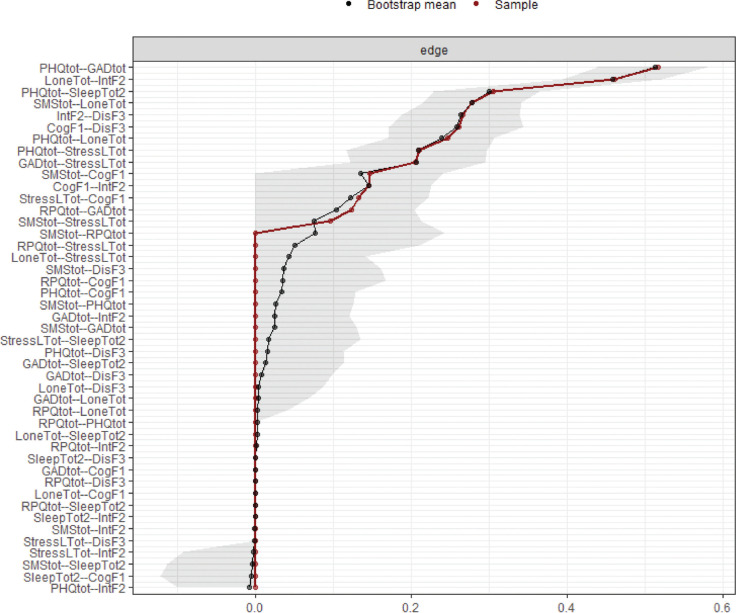
Bootstrapped CIs of estimated edge-weights for the estimated network at time 3. The red line indicates the sample values and the grey area indicates the bootstrapped CIs. Each horizontal line represents one edge of the network ordered by edge-weights.

**Figure S8:**
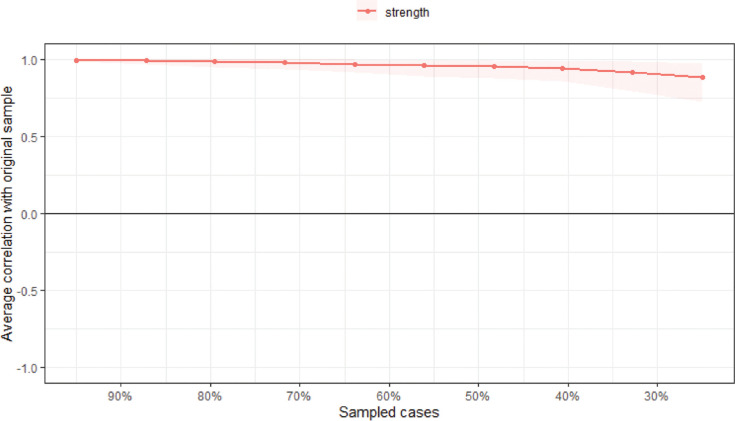
Average correlations between strengths of networks estimated for time 3 with sampled participants and original sample. Lines indicate the means and areas indicate the range from the 2.5th to the 97.5th percentile. The CS-coefficient for strength [CS (cor = 0.7) = 0.751] is higher than 0.25 suggesting the centrality indices were relatively stable.

## Data Availability

The datasets generated during and/or analysed during the current study are available in the repository: http://www.doi.org/10.5522/04/16583861

## Notes

1 Dimensions Covid-19 database. https://reports.dimensions.ai/covid-19/

2 UCL COVID Social Study. https://www.covidsocialstudy.org/

3 Data comes from Wikipedia, government health ministries, *The New York Times*, and other authoritative sources, as attributed as of 14 April 2022.

## Network stability and accuracy

Bootstrapping with 2500 permutations was performed to estimate the accuracy of edge-weights. Bootstrapped CIs are plotted in Fig. S1. The relatively narrow bootstrapped CIs suggested that the order of the edges in the network was stable.

The stability of the order of centrality indices was investigated based on observation of subsets of the data (2500 permutations). Figure S2 below shows the good stability of strength. Stability of centrality indices could be quantified using the **CS-coefficient**, which calculated the maximum drop in proportions to retain a correlation of 0.7 in at least 95% of the sample. We found that the CS-coefficient for strength (CS (cor = 0.7) = 0.75) is higher than 0.5 suggesting the centrality indices were stable.

We then performed bootstrapped difference tests (with 2500 permutations) of edge-weights and centrality indices to test whether they differed significantly from each other. The results are shown in Figs S3 and S4, respectively.

Figure S5–S6 shows stability of edge-weights and centrality for estimated network for wave 2; and figure S7–S8 shows stability of edge-weights and centrality for estimated network for wave 3.
